# Advanced CNN Architecture for Brain Tumor Segmentation and Classification using BraTS-GOAT 2024 Dataset

**DOI:** 10.2174/0115734056344235241217155930

**Published:** 2025-01-02

**Authors:** Vaidehi Satushe, Vibha Vyas, Shilpa Metkar, Davinder Paul Singh

**Affiliations:** 1 Department of Electronics & Telecommunication Engineering, COEP Technological University (COEP Tech), Pune, Maharashtra, India; 2 Department of Computer Science and Engineering, Pandit Deen Dayal Energy University, Gujarat, Gandhinagar, India

**Keywords:** Brain tumor, Generalizability, CNN, MRI, AGI, BraTS-GoAT

## Abstract

**Background:**

The BraTS Generalizability Across Tumors (BraTS-GoAT) initiative addresses the critical need for robust and generalizable models in brain tumor segmentation. Despite advancements in automated segmentation techniques, the variability in tumor characteristics and imaging modalities across clinical settings presents a significant challenge.

**Objective:**

This study aims to develop an advanced CNN-based model for brain tumor segmentation that enhances consistency and utility across diverse clinical environments. The objective is to improve the generalizability of CNN models by applying them to large-scale datasets and integrating robust preprocessing techniques.

**Methods:**

The proposed approach involves the application of advanced CNN models to the BraTS 2024 challenge dataset, incorporating preprocessing techniques such as standardization, feature extraction, and segmentation. The model's performance was evaluated based on accuracy, mean Intersection over Union (IOU), average Dice coefficient, Hausdorff 95 score, precision, sensitivity, and specificity.

**Results:**

The model achieved an accuracy of 98.47%, a mean IOU of 0.8185, an average Dice coefficient of 0.7, an average Hausdorff 95 score of 1.66, a precision of 98.55%, a sensitivity of 98.40%, and a specificity of 99.52%. These results demonstrate a significant improvement over the current gold standard in brain tumor segmentation.

**Conclusion:**

The findings of this study contribute to establishing benchmarks for generalizability in medical imaging, promoting the adoption of CNN-based brain tumor segmentation models in diverse clinical environments. This work has the potential to improve outcomes for patients with brain tumors by enhancing the reliability and effectiveness of automated segmentation techniques.

## 1 INTRODUCTION

Segmenting a brain tumor is an important part of medical prediction because it gives doctors important information for planning treatments and keeping an eye on things. Imaging scans from magnetic resonance imaging (MRI) are often used for this [1, 2]. Precise and environmentally friendly segmen-tation is crucial for accurately identifying tumor borders, enabling doctors to make informed decisions. Brain tumors develop when cells undergo aberrant and uncontrolled proliferation. The most prevalent kind of tumors are the ones that originate in the brain, referred to as “primary.” Tumors from other parts of the body have grown in many places here. This is another name for secondary cancers. That type of cancer doesn't spread. Every growth does have a chance of keeping safe, though, or it could turn into cancer. Any kind of growth is dangerous, though. The surface area of the human brain is limited, and the growth of cancerous tumors increases the risk to the patient's life [3]. When intracranial pressure goes up, blood flow to the brain slows down. Normal organs can swell and cells can get damaged by this. Multiple kinds of brain tumors exist, and all of them can be very dangerous. Glioma is the most common type of growth that can be handled and usually doesn't last. As far as types go, there are two main kinds of gliomas: high-grade and low-grade. HGG is very dangerous because it grows so quickly compared to LGG. An updated study of a Canadian population from 2009 to 2013 using data from the central nervous system (CNS) [4] found that people with HGG aged 20 to 44 were still alive after 14 months of treatment. See Fig. (**1**) for an example of how the life expectancy varies for various types of brain tumors.

Magnetic resonance pictures (MRIs) are the best way to tell brain tumors apart from other types of tumors. They give you the benefits of both visual analysis and a lot of freedom when using computers to look at medical pictures. It is possible to find cancer with medical imaging tests like MRIs, CT scans, Positron emission tomography (PET), and more. These methods help find the growth and check to see how far it has grown before and after treatment. MRI, or magnetic resonance imaging, is often the best way to find brain cancers and figure out how to treat them [6]. It doesn't hurt the patient, makes high-quality pictures, and can tell the difference between hard and soft tissues. Brain tumors are usually best treated with surgery, but drugs and radiation may also be used to arrest the tumor's growth [7]. To see different parts of the brain, you need a lot of MRI slices, such as FLAIR, T1, T2, and T1 contrast pictures.

World Health Organization (WHO) divides brain tumors into four groups based on how fast they are growing: grade I, II, III, and IV. The least dangerous tumors are Grade I ones, which means they are not cancerous at all. Grade I tumors, however, may pose a significant risk to life even in cases when surgery is not feasible. Tumors of grade II progress at a faster rate and have a more abnormal microscopic appearance compared to grade I tumors. Tumors of this grade have the capacity to invade adjacent healthy tissue and reemerge as malignancies of grade III or higher. Tumors at this stage are classified as malignant. These tumors have a high tendency to recur. Grade IV tumors are very malignant since they have the propensity to infiltrate a significant area of adjacent normal tissue. Radiation treatment is applied to the specific areas of the brain that are impacted by the tumor [8].

The importance of brain tumor segmentation can never be overstated in the field of medical imaging as it helps in diagnostics and treatment planning. Image segmentation can be defined as the separation of an image into several sub-parts according to the defined criteria such as intensity, texture, or position. When discussing tumor segmentation, entails the identification of a tumor and its contouring within medical imaging such as MRI scans which are crucial in diagnosis and treatment.

There are several problems that come up in relation to such tasks, and these include the heterogeneity in the shape, location, and size of the tumor in different patients and the noise or artifacts that are present in the medical images. There is also the problem of achieving generality among many different types of tumors, and many imaging protocols as well [9-11]. To solve such problems, advanced methods based on Convolutional Neural Networks (CNNs) have been created since CNNs have the potential for image feature extraction, spatial hierarchy learning, and performing better in segmentation.

This study presents a new framework that goes by the name of BraTS-GoAT. It seeks to improve the brain tumor segmentation models such that they can work well across different types of tumors. The approach is able to address the constraints posed by these defining features by concentrating on the learning of invariant features over classes of different tumors, within constraints of being tumor class specific. This enables performing tumor segmentation tasks more effectively, thus making it usable in practice where accurate segmentation is a prerequisite for treatment planning and predicting clinical outcomes.

The aim of the paper in its current format is to develop an advanced CNN-based model for brain tumor segmentation using the BraTS-GOAT 2024 dataset. The contribution of our study is as follows:

### 1.1 BraTS-GoAT Framework

The suggested method overcomes brain tumor segmen-tation difficulties by sharing features that are invariant to different types of tumors while being able to maintain tumor-specific features, which facilitates better robustness and performance across different tumor types and imaging modalities.

### 1.2 State-of-the-art Performance

The method performs better than other existing approaches in terms of various evaluation metrics such as Dice score, precision, accuracy, and sensitivity, with the authors having provided better segmentation results as compared with other methods specified earlier in the literature.

### 1.3 Enhanced Generalizability

Focusing on generalizability, BraTS-GoAT makes sure that the model will be able to apply to a variety of shapes, sizes, and even locations of tumors, thereby making it fitting across a number of clinical scenarios as well as MRI protocols.

### 1.4 Clinical Relevance

There is a considerable potential for the framework in clinical settings where segmentation is necessary for correct treatment and for the monitoring of the progress of the tumor. The study proposes a future outlook of the combination of radiomics features from the segmented tumors integrating clinical information that can make personalized treatment approaches more effective.

The Novelty of our study is as follows:

- The novelty of this work lies in its generalizable tumor segmentation approach that combines robust perfor-mance with clinical applicability, paving the way for future advancements in personalized medicine for brain tumor detection and treatment.

The exact structure of this paper looks like this: The third section discusses a review of the literature. The fourth section discusses the method. The fifth section discusses the study's results. And finally, the sixth section discusses the study's conclusion.

## 2 BRAIN TUMOR SEGMENTATION

**Fig. (2):**
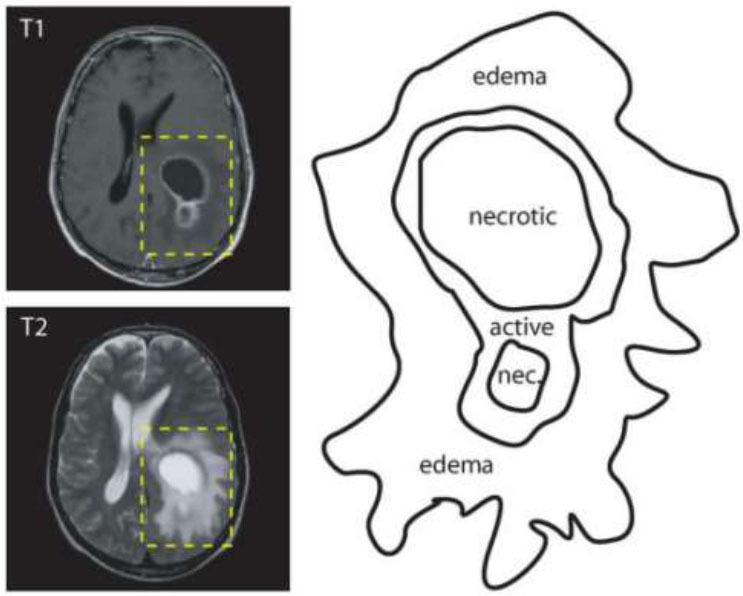
Labeled example of a brain tumor illustrating the importance of the different modalities [9].

There is a process called brain tumor segmentation that is needed to properly find and outline tumor areas in brain scans. These scans are usually done with MRI or CT pictures. For a correct diagnosis, to plan effective treatment, and to keep an eye on how the illness is getting worse or better, this process is necessary. Precise delineation of brain tumors may have a substantial influence on patient outcomes by facilitating meticulous surgical preparation, focused radiation therapy, and monitoring of tumor response to treatment. The segmentation procedure may be very difficult owing to the intricate structure of brain anatomy and the variety in tumor form, size, and placement. Tumors may have asymmetrical forms, and their borders may be indistinct, merging with adjacent tissues. Furthermore, many categories of brain tumors, including gliomas, meningiomas, and metastases, display distinct imaging features, which adds complexity to the process of segmenting them. This part gives a quick overview of brain tumor division. Partitioning brain tumors into segments is done to find the exact location and size of the tumor (Fig. **2**): Active tumorous tissue, Necrotic (dead) tissue, and Edema (Swelling near the tumor). In Fig. (**2**), two types of MRI scans—T1 with contrast and T2—were used to clearly show where the tumors were.

## 3 REVIEW OF LITERATURE

This part talks about different brain tumor segmentation methods and improvements, focused on how strong and useful models are for different types of tumors and imaging methods.

Jiajia Li., (2024) [12] uses the denoising diffusion of diffusions to generate some primary segmentation results DiffCAS. During the process of denoising diffusion, semantic information is non-invasively extracted by utilizing Swin Transformer for CTA images and they propose an adaptive residual feature enhancement (ARFE) module as denoising encoder in the diffusion model, a feature fusion attention (FFA) module unifies features from Swim-Transformer with denosing encoders enhancing segmentation performance. DiffCAS achieves state-of-the-art performance in terms of the Dice coefficients 84.41% and 84.59%, on the ASOCA dataset and ImageCAS dataset, respectively.

Sunil *et al*., (2024) [13] studied the development of integrating Machine Learning (ML) with clinical imaging diagnostics to increase AGI or Artificial General Intelligence, which is now being worked on. An MRI scan can reveal a lot about the biology of brain tumors, which changes the decision of whether to perform invasive surgery. But even experts who use MRIs don't always get it right; this mistake rate can reach 85%. It is a shame that the best machine-learning models for brain tumors have problems with learning too fast and fitting the data too well. Large models with lots of moving parts show these issues more clearly. SIENNA, a deep machine learning (ML) model for finding brain tumors, did better than some other state-of-the-art (SOTA) models in four key ways. A better design that lowers overfitting and speeds up learning is used in this work, along with a mixed inductive cross-validation method that makes the results more general. In this case, SIENNA can connect to both 1.5 Tesla and 3.0 Tesla MRI machines. Clinical DICOM MRI data can be roughly divided into three groups based on how accurate they are: non-tumor (92% accuracy), GBM (91% accuracy), and MET (93% accuracy). F1 and AUROC scores that are high help SIENNA keep the number of false positives and rejections low. Sienna is a streamlined and improved system for artificial general intelligence (AGI) that is meant to be used in medical settings. It can also work with new data sources that include different types of information without any problems.

Kim *et al*., (2024) [14] developed a framework that lowers the amount of data required while still retaining performance. Picture Three-dimensional U-net models were trained on MRIs of 638 brain tumors from several schools, and different active learning methods were tested. For both training and testing, the results were about the same with the Bayesian estimate with dropout as with the full data model. A random question only needed about 30% of the training data to perform at the expected level (*p* = 0.018). This was achievable. By 20%, methods that stop comments from being used more than once might lower the amount of training data that random queries need to succeed. They looked at a number of active learning methods that could cut down on the amount of human labeling that was needed to divide brain tumors into three-dimensional segments. The estimate of dropout uncertainty successfully reached the desired level of performance using the minimum amount of annotated data.

Berkley *et al*., (2023) [15] assessed the suitability and transferability of cutting-edge deep learning models using novel clinical data across different institutions. They used a cutting-edge 3D U-Net model to train on the traditional BraTS dataset, which consists of both low and high grade gliomas. Then, they used the clinical data to see how well the method worked at instantly telling the difference between brain tumors. This collection has more kinds of tumors, sizes, and ways to rate them than the BraTS collection. It is common for clinical MRIs to show Dice scores of 0.764 for the biggest tumor, 0.648 for the core of the tumor, and 0.61 for the enhancing tumor. For both the same institution and cross-institution datasets from different sources and gathered in different ways, these values are higher than the numbers that were previously publicly available.

Hu *et al*., (2023) [16] studied improvements in deep learning for brain tumor segmentation have shown good results when the distribution of the training and test data is similar. The way medical pictures are shared can change because of different types of cancer, the way images are made, and data from different medical sites. The study's goal is to make the model better at generalizing by making clear changes to it within a group. With the Mixture of Calibrated Networks (MCN) method, a lot of networks are trained on both the original and better regions at the same time. When we test the expectation-maximization (EM) method, we find out more about the parameters and see how the important parameters are linked. We can then better understand and guess what the conditions will be. It was constantly easier to use what was learned in new situations, and the suggested method was more accurate at predicting results than both approaches that work in different domains and approaches that work in the same domain.

Koirala *et al*., (2023) [17] investigated the use of deep learning techniques to improve the accuracy of brain tumor segmentation in Sub-Saharan African patients by analyzing multi-modality magnetic resonance imaging (MRI) data. You can choose from eleven different forms of their ensemble method, which is based on three main architectures: UNet3D, ONet3D, and SphereNet3D. Each version has a different loss function. Understanding how the brain works requires segmentation models that look at both age and social factors, as this study showed. Based on the study's findings, using an ensemble method with multiple patterns is more effective than using a single model, which in turn leads to better assessment tools. According to the dice, the words “enhancing tumor,” “tumor core,” and “total tumor” all have numbers of 0.82. These results show that personalized deep-learning methods can correctly divide brain tumors into segments. They also lay the groundwork for future research that will improve models and test how well they work in other parts of the brain.

Stember *et al*., (2022) [18] studied that supervised deep learning in radiology is hindered by well-known intrinsic limitations: firstly, it requires extensive, manually labeled datasets; secondly, it lacks the capacity to generalize; and thirdly, it lacks explainability and intuitive. Recently, it was suggested that reinforcement learning might be able to solve all three of these issues. Researchers used data from doctors' eye tracking and deep reinforcement learning to correctly find where brain tumors are. It's not perfect because it limits the states and actions that can happen. A Deep Neural Network was taught using 30 two-dimensional images from the BraTS brain tumor library. There was only one growth in each picture. Later, they used a different set of 30 pictures to test the Deep Q network that had been trained. Guided learning was used to test a deep learning network that can find important data. This network used the same set of pictures as the last one to train and test itself. The guided strategy quickly got too good at fitting the training data and always did badly on the testing set (with an accuracy of 11%). On the other hand, the Deep Q learning method got better over time at generalizing to the testing set and reached a 100% success rate. They have effectively used reinforcement learning to accurately identify the location of brain cancers using 2D contrast-enhanced MRI brain images. They have shown that reinforcement learning has generalization capabilities to a distinct testing set and does not suffer from overfitting when trained on small datasets.

Rawat *et al*., (2022) [19] evaluated the effectiveness of the layer normalization approach in a CNN-based 3D U-Net for improving training efficiency and enhancing generalization in the field of medical imaging. Language Normalization and Recurrent Neural Networks are often used together in Natural Language Processing jobs like making written patterns and asking questions. Ways LN can be used in medical imaging need more research. It was learned with and without LN (local normalization) using brain MRI segmentation as part of this method. It was found that the LN-based model has less validity loss than the model that wasn't adjusted when they put them next to each other. When unknown data was sent to the LN-based model before normalization, the validation dice scores were 0.90 for edema, 0.74 for tumors that weren't enhancing, and 0.95 for tumors that were enhancing. It's better to have these numbers than not having them normalized.

Henry *et al*., (2021) [20] Segmenting brain tumors is a vital task for effectively managing a patient's condition. They used many U-net-inspired neural networks to simplify and standardize this process. These networks were mostly trained with random weight averaging and deep guidance methods. This challenge used data from the BraTS 2020 training set to help with the Multimodal Brain Tumor Segmentation. To make a picture showing where brain tumors are, different groups of models were taught in different ways. The labels from both groups of patients were looked at, and then the results from some growth areas were put together. As the test time was raised, the following findings showed that the online review dataset worked: With a 95% chance of being correct, the Hausdorff distances between the enhancing tumor, the whole tumor, the tumor core, and the other two parts are 20.6 mm, 4 mm, (3 mm), and 5.7 mm. 0.81, 0.91, and 0.85 are the dice coefficients for the tumor parts. The end test dataset following the suggested method had a Dice coefficient of 0.79, a Hausdorff distance (95%) of 20.4, a 6.7 mm, and a 19.5 mm.

Divya *et al*., (2021) [21] created very efficient encoder-decoder architectures to automatically separate brain cancers from low-resolution 2D pictures. Brain tumor segmentation works better when the Ensemble of Multiple Architectures (EMMA) is used. The computers that run these models are also faster than the ones that were used in the BraTS challenge. It takes 0.82 Fl-scores for the Tumor Core, 0.87 Fl-scores for Whole Tumor, and 0.78 Fl-scores for Enhancing Tumor to solve one of the BraTS challenge datasets. The KMC-Manipal dataset has average Fl-scores of 0.74 for TC, 0.82 for WT, and 0.68 for ET.

Hua *et al*., (2020) [22] introduced a new approach called cascaded V-Nets to accurately identify and separate different parts of brain tumors in multimodal brain magnetic resonance imaging. They showed that V-Net could do even better by adding a cascaded structure and the ensemble method. It has already done well in a number of category tasks. They showed three different ways to make mixed magnetic resonance pictures so that different models could be trained. It's better when you mix segmentation probability maps from various models. The doctors advised cutting the growth into three sections, one for each of the three stages: dying, swelling, and growing. As tested on the BraTS 2018 online validation set, the average Dice scores for the general tumor, the tumor core, and the boosting tumor are 0.9048, 0.8364, and 0.7748. At the online BraTS 2018 test, people got scores of 0.8761, 0.7953, and 0.7364.

Xue *et al*., (2020) [23] examined an accurate determination of brain tumor type, morphology, and dimensions using MRI images is crucial for the diagnosis and treatment of glioma. It can be hard to avoid making mistakes when you search for growth by hand. We now have convolutional U-Net designs, which are made up of encoders and decoders, which are the most advanced and useful automatic systems for picture segmentation. A convolutional U-Net system with various encoders for every kind of picture was the focus of the research. Various fusion and decoding blocks receive the outputs from the encoders for each part of the tumor.

Wang *et al*., (2019) [24] presented a series of Convo-lutional Neural Networks (CNNs) to divide brain tumors into hierarchical subregions using multi-modal Magnetic Reso-nance Imaging. Also, they made a 2.5D network that does a good job of balancing model complexity, memory use, and receptive field. The study showed that the BraTS 2017 dataset worked really well with the cascaded approach using 2.5D CNNs. In the BraTS challenge, it was the second-best method that was used. In addition, they used the BraTS 2018 dataset to make sure that the method worked. The results showed that adding test-time enrichment makes brain tumor segmentation more accurate. In addition, the unknown information gathered from this method may help find likely mistakes in segmentation and make segmentation even more accurate. There were many writers who worked on the BraTS (BraTS) Challenge. Table **1** shows what they found.

## 4 RESEARCH METHODOLOGY

This section talks about the steps that were taken to make the system. It includes details about the final layout and the unique features of the suggested system. Fig. (**3**) shows a rough sketch of how the suggested system is set up. Using deep learning to look at medical images has been very helpful, especially for sorting brain tumors into different groups. The methods are tested twice to make sure that the CNN model is reliable and good at finding new MRI pictures. There are two key parts to the process flow: preparing images and training CNN models. To get around the problems that come with preprocessing methods not being regular, the system shown in Fig. (**3**) combines important features that give reliable and consistent results. The core of our approach revolves around the following techniques.

The steps of flowchart in Fig. (**4**) are described below.

### 4.1 Step 1: Dataset

An MRI study of the brain is the first step. MRI stands for magnetic resonance imaging. It is a test. The screening method doesn't hurt the person and clearly shows the brain's soft tissues, which is very helpful for finding problems like tumors. A variety of populations, including adults, children, and underrepresented groups from sub-Saharan Africa, were used to build the information for this challenge. There are 360 scans in the validation set and 2,251 scans in the training set of brain MRIs. T1, T1Gd, T2, and T2-FLAIR are the MRI modalities that are offered. Three tumor subtypes are identified by the expert annotations included with each scan: enhancing tumor (ET), tumor core (TC), and entire tumor (WT). It's interesting to remember that malignancies can be seen in any MRI scan. Every MRI scan is always 240 × 240 x 155 in size. Expert neuroradiologists annotated and approved all of the training data. The enhancing tumor (ET - label 3), necrotic tumor core (NCR - label 1), and peritumoral edematous/invasive tissue (ED - label 2) are the labeling values. The data are pre-processed, meaning they have previously been skull-stripped, co-registered to the same anatomical template, and interpolated to the same resolution (1 mm^3^), just like in prior editions. No external data is allowed in order to maintain competition

### 4.2 Step 2: Data Pre-processing

Medical image analysis depends on image preparation results a lot for accurate understanding and analysis. The goal of image preparation is to improve picture quality, lower noise, and pull out important features. Fig. (**4**) shows that the many steps we take in the preparation step are meant to make the pictures we start with better and more regular. The first thing we do is use Formula 1 to improve the picture's color and quality by equalizing the histogram.

The contrast of a picture is a way to measure how much local difference there is.

**Table:** 

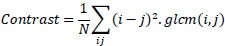

This approach enables enhanced viewing of structures and imperfections. We then use median filtering to smooth out the picture and lower the noise, which makes the patterns clearer. Using a method called contrast-limited adaptive histogram equalization, the suggested plan raises the contrast even more. This approach effectively mitigates the excessive amplification of noise while maintaining a uniform improvement of local contrast over the whole image.

### 4.3 Step 3: Feature Extraction

Once the images are preprocessed, the next step is featuring extraction, which involves analyzing the images to identify significant patterns and characteristics. This step includes:

#### 4.3.1 Texture Analysis

This method evaluates the texture of the brain tissue, capturing patterns such as smoothness, roughness, and granularity. Texture features are crucial for distinguishing between different types of tissues and abnormalities. A lot of people use the GLCM to look at textures. You can use the GLCM to make a number of statistical metrics that measure different aspects of the material.

To construct a GLCM, we use the following formula:

P(i,j|d,θ)P(i, j | d, θ)

Where:

- P(i,j|d,θ) is the chance that the two gray levels i and j will be d apart in the direction ¸.
- Its strength numbers, or gray levels, are i and j.
- d is the distance between two pixels.
- The angle (°) between the two pixels. Angles 0°, 45°, 90°, and 135° are used a lot.

#### 4.3.2 Shape Analysis

This involves analyzing the shapes and contours of structures within the brain. Shape features help in identifying the geometric properties of tumors, such as their size, perimeter, and irregularity. By incorporating important mathematical traits, these qualities help to identify different types of tumors.

The merged features extracted from GLCM (Gray-Level Co-occurrence Matrix) and shape contours used in this paper can be considered as a way to fuse texture information with structural characteristics within a CNN model. Texture features that GLCM provides (contrast, correlation, energy) assist in recognizing the image pattern. Shape contours that describe the boundaries of an object (*e.g*, a tumor) and provide information about its overall shape and location. These can be collected in parallel with the image into a dual-branch network composed of one branch for raw data and another oscillating between GLCM or contour features. The CNN features are then used to extract these features domestically and the same are extracted with those from dumped models,, further fused for good segmentation or classification. It works well for tasks like brain tumor segmentation since texture + shape are important contributions. The CNN with GLCM and contour of the shape features show better results in segmentation compared to using just the CNN. They do not provide texture information of the image to distinguish among different tissues, although they can enhance tumor boundary delineation through shape contour features.

### 4.4 Step 4: Classification using CNN

The traits that were taken are then sent to a (CNN) so that it can classify the data. A big set of labeled MRI pictures is used to train the CNN. This set has both training data and testing data:

#### 4.4.1 Training Data

This dataset is used to train the CNN model. It includes MRI images with known tumor types and annotations. During training, CNN learns to spot trends and traits that are linked to different kinds of brain cancer.

#### 4.4.2 Testing Data

This set of data is used to test how well the learned CNN model works. It has MRI pictures that the model has never seen before. The model's memory, accuracy, precision, and dice score are all checked to make sure it works well with new data that it hasn't seen before.

### 4.5 Step 5: Identify the Type of Brain Tumor

The final step involves using the trained CNN model to detect and classify brain tumors in new MRI images. The model analyzes the input MRI images, identifies regions that likely contain tumors, and classifies the type of tumor based on the learned features.

### 4.6 Technique

#### 4.6.1 CNN

**Fig. (6):**
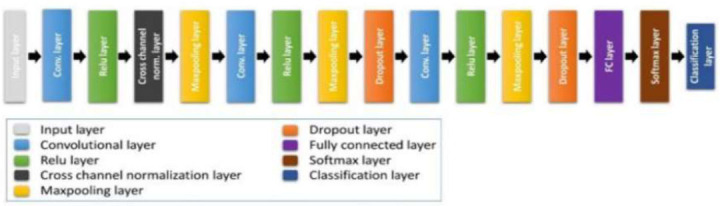
Architecture of CNN.

(CNN) is a great deep-learning technology that is often used to find brain tumors in MRI images and put them into groups [25]. Convolutional neural networks (CNNs) are made to instantly and easily learn spatial hierarchies of charac-teristics from received pictures. Assisting in this task are several fully linked, convolutional, and pooling layers. The Convolutional Neural Network (CNN) uses MRI images to study brain tumors. It does this by first “convolutionalizing” the images to find edges, surfaces, and patterns [26]. These things are very important for telling the difference between different types of tissue. The next pooling levels reduce the number of spatial dimensions, which helps focus on the most important features while easing the computational load. The traits are then sent to fully connected layers, which look at the patterns to decide if there is a brain tumor and what kind it is, like gliomas, meningiomas, or other types. This method is very helpful because it can effectively handle the complicated and uncertain shapes and textures of brain tumors. It instantly finds tumors very accurately and reliably, which is very important for early detection and planning treatment for patient (Fig. (**5**). (Fig. **6**) depicts the architecture of CNN.

### 4.7 Encoder-decoder Architecture

#### 4.7.1 Encoder

The encoder is formed of a number of convolutional layers and pooling operations that extract features from the input medical images (MRI scans) in hierarchically. At higher layers of the encoder, it reduces the spatial input dimensions slowly to represent abstract features.

#### 4.7.2 Decoder

Feature maps are up-sampled to the spatial resolution using deconvolution (transpose convolution) layers; a final segmen-tation map is generated of input image size.

#### 4.7.3 Skip Connections

They thus keep those connections between the encoder and decoder to continue giving fine-shot spatial information that is lost through down-sampling, ending with better segmentation map accuracy. This can be particularly helpful for tasks such as accurate segmentation of tumor boundaries *e.g*. in brain tumor segmentation.

### 4.8 Pixel-level Classification

CNNs for pixel-level classification output a label in an input image so that each pixel is labeled as tumor, edema, healthy tissue *etc*. The network outputs a segmentation mask, with each pixel classified as shedding to the tumor region or other anatomic structures.

### 4.9 Activation: Softmax or Sigmoid

For multi-class segmentation tasks, a softmax activation function is often used in the last layer to get the probability of each class for the respective pixel. In the case of binary segmentation (tumor *vs*. non-tumor), a sigmoid activation is applied to achieve an output probability map for each pixel.

#### 4.9.1 Loss Function

The most common loss function used to optimize the network is Dice Loss (also known as cross-entropy loss). Dice Loss is especially useful in the case of imbalanced datasets(usually found for medical imaging) where background pixels are far more than the number of tumor regions.

A Non-enhanced T1, a contrast-agent coregistered FLAIR and Post Contrast-Enhancing tumor labels are necessary in most current brain tumor segmentation tasks due to these possible multi-class groups:

Background- the non-tumor-containing regions in the brain (normal tissue).Edema (ET): The area of the tumor that enhances on imaging, often related to an edema or swelling around the tumor. Tumor Core (TC): the core of the tumor, regardless if it has enhancement.Whole Tumor (WT): The entire tumor region, which contains the enhancing tumor (ET), as well as any necrotic tissue that is part of the indeed present lesion and tumor core. These categories allow to determine the overall architecture of a tumor, which can deliver important insights into its diagnosis, therapy, and tracking progression body.

## 5 RESULTS AND DISCUSSION

In this research, we tested how well our suggested model could classify brain tumors by looking at its accuracy, precision, memory, and Dice score, which are all explained below. The model that learned to spot different kinds of brain tumors' report on how they were classified is shown in Table **2**. It shows things like the F1 score, precision, memory, and help for each class. The indicator “NOT Tumor” is very good at finding places that aren't tumors, as shown by its high F1-score of 0.99 and high accuracy. There are a lot of examples, 437,509,975, that support this description. The “NCR” (non-enhancing core) has shown average accuracy (0.35), memory (0.38), and F1-score (0.36) in a total of 471,935 cases, suggesting average ability. With a score of 0.24 for precision, 0.37 for memory, and 0.29 for F1 score, the word “ED” (edema) does not do as well as it could. It has been used 2,907,516 times. The “ET” (enhancing tumor) has an F1-score of 0.42, a precision of 0.67, and a recall of 0.30 for a total of 1,478,574 cases. It is clear from these tests that the model is more accurate but less able to remember things. An F1-score of 0.52 means that the model does well in all classes. It is accurate 0.98 times, has an average precision of 0.56 times, and recalls 0.51. The model does a good job overall, as shown by the high accuracy (0.99), recall (0.98), and F1-score (0.99) of the weighted average that takes support into account. The main reason for this is the large number of cases that are not linked to tumors.

**Fig. (7):**
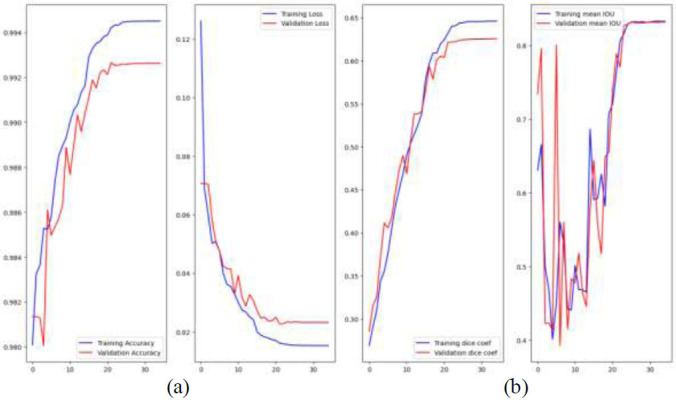
(**a**) Training and validation of accuracy and loss, (**b**) Dice coeff and Mean IOU of training and validation.

Fig. (**7**) shows four plots that show how well a deep learning model did during training and evaluation over 30 iterations. It can be seen in the first picture in Fig. (**7a**). The model is getting better because both the training and confirmation accuracy keep going up. The accuracy for training is a little better than the accuracy for confirmation. The loss is shown in the second figure. The model is learning well because both the training loss and the validation loss go down over time. However, the training loss is lower, which could mean that it is too good at what it does. It can be seen in the third curve in Fig. (**7b**). Both the training and validation Dice coefficients are going up, which means that the expected and real segments are more likely to overlap, and they are staying close to each other, which means that the model works well in most situations. The fourth graph displays the average Intersection over Union (IoU). Both the training and validation IoU go up, but there is more variation in the variability, especially in the validation set. This suggests that the validation performance isn't stable, but that the model's accuracy and segmentation quality are generally getting better. Table **3** shows that Adam Optimizer is used to train neural networks. Softmax activation function is used at the output layer for classification. Categorical cross entropy is used as a loss function for the multiclass-classification problem. (Fig. **7**).

Fig. (**8**) depcit the performance metrics of testing data as shown below. From this Fig. (**7**), the proposed model attained the accuracy of 0.9847, mean IOU of 0.8185, dice coef of 0.3734, precision of 0.9855, sensitivity of 0.9840, specificity of 0.9952, dice coef edema of 0.2086, and dice coef enhancing of 0.2919 as shown below.

Fig. (**8**) shows how well a machine learning model worked at telling the difference between different parts of tumors in MRI pictures. The Enhancing Tumor (ET), the Whole Tumor (WT), and the Tumor Core (TC) are some of these. It is possible to measure two important variables: the Dice coefficient and the Hausdorff distance. Fig. (**9**) shows the performance metrics of the proposed method. Both WT and TC got a score of 0.8740 on the dice, which means that they were properly split up. ET's Dice score, on the other hand, is 0.4959, which shows it wasn't divided as well. Both WT and TC have Hausdorff distances of 1.4142, which means that the segmentation edges of WT and TC are pretty close to the ground truth. However, the Hausdorff distance for ET is 2.2361, showing that the segmentation boundaries for ET are less accurate. These metrics indicate that the model is effective in segmenting WT and TC, but there is still space for improvement in reliably segmenting ET.

Based on two evaluation criteria, the Dice Coefficient and the Hausdorff Distance, Fig. (**10**) shows how well a segmentation model works. In dice coefficient statistics, the data shows that the average overlap is moderate, with a mean of 0.7493. The performance varies, as indicated by the standard deviation (STD Dev.) of 0.1774. Half of the predictions have an overlap better than the median value of 0.8748. Additionally, 25% of predictions have an overlap below the 25th percentile value of 0.6866, while 75% of predictions have an overlap below the 75th percentile value of 0.8748. In the Hausdorff Distance statistics, the mean distance is -8.3118, indicating an average underestimation. The standard deviation is 13.7547, showing high variability. The median distance is 1.4142, suggesting that half of the predictions have a distance better than this value. The 25^th^ percentile is -13.1749, indicating that 25% of predictions have a distance worse than this value. The 75th percentile is 1.4142, indicating that 75% of predictions have a distance below this value. The presence of a negative mean and 25th percentile in the Hausdorff Distance may indicate a mistake in the computation or interpretation since distances are generally considered to be non-negative.

Fig. (**10**) shows two brain MRI images obtained using the FLAIR (Fluid-Attenuated Inversion Recovery) imaging technique. The picture on the left displays the unprocessed FLAIR scan, which accentuates areas of the brain with irregularities by rendering cerebrospinal fluid (CSF) black. The right picture shows a FLAIR scan that has been enhanced using an overlay mask. This mask is probably used to make it easy to see certain areas of interest, like sores or spots where a disease has had an effect. The overlay mask facilitates the visualization and quantification of brain abnormalities. Table **3** gives us the hyperparameter values of the proposed model which states that a solid training setup is provided for configuring a neural network model to perform brain tumor segmentation and classification. The Adam optimizer that the model will be trained over 200 epochs with has been widely recognized for its efficacy in addition to being adaptive. This type of activation function is well-suited to multi-class classification tasks and so the softmax activation function is used in this case. For the chosen loss function, categorical cross-entropy can be employed as it measures the performance of a probabilistic classification model *i.e*., one whose output ranges between 0 and 1. To evaluate how this model performs, metrics such as accuracy, Mean Intersection over Union (MeanIoU), and Dice coefficient are introduced to provide a holistic appraisal of how precise our model is in comparison with the true labels. Callback functions like CSV logger, early stopping, and ModelCheckpoint are integrated into this system so as to help improve its training process. These callbacks are essential in monitoring training progress, avoiding overfitting by stopping early when there’s no improvement noticed, and saving best model weights during the course of training.

Fig. (**11**) displays the segmentation mask of the FLAIR image. The multi-class images of segmentation masks indicate the place and limits of different tissues or disorders like tumors.

The model is tested using a training call-back and a batch size of 100. Fig. (**12**) shows that the OEDEMA forecast is 98.24 percent accurate when compared to the ground reality. Pictured below are two segmentation maps associated with brain imaging, side by side for your perusal. The identification of oedema is the primary emphasis of the comparison. The “ground truth” is shown on the left margin of the figure. This “ground truth” represents the oedema-related manually-marked or recognized points of importance. The white spots on the dark background in this image reveal exactly where the oedema is in the brain slice. The class represented by the letter “OEDEMA” on the right is the one for which this model is making its prediction. The fact that the predicted areas are shown as different Greyscale tones on a black backdrop implies that each pixel in the model generates a probabilistic result, with darker regions being less likely and brighter regions being more likely. The ground truth has clear and distinct zones, whereas the forecast is more diffuse and unclear.

### 5.1 Masking

The function np.ma.masked where is used to create a masked array such that a condition for example mask value equals 0 will be used to hide some parts of the mask. This way only relevant parts of the mask will be shown.Overlaying: The original MRI image is overlaid with transparency (alpha parameter) so that both anatomical structure and segmented regions can be visualized at once. Fig. (**13**) displays a sequence of brain MRI scans with FLAIR imaging, depicting several phases of processing. The first picture is the unaltered FLAIR scan. The second picture depicts the ground truth, which is a carefully annotated reference that identifies regions of interest, such as anomalies or lesions. The third picture displays the superimposed representation of all expected anomalies from various classes on the original scan. A different color stands for each type of tumor. The next three pictures show how the different types of lesions should be divided: NCR (non-enhancing core), ED, and ET. Color-coding the expected segments makes it easier to see which brain areas go with them, which makes it easier to compare the real data with the model's predictions. Table **4**. shows a comparison of the different performance metrices of the proposed approach against some methods in the literature. Except for the Dice coefficient of Enhanced tumor, all other metrics show better results when compared with other methods.

## CONCLUSION AND FUTURE WORK

In this study, we have introduced BraTS-GoAT, a novel framework aimed at enhancing the generalizability of Brain Tumor Segmentation (BraTS) models across diverse tumor types. Through comprehensive experimentation and analysis, we have demonstrated the effectiveness of BraTS-GoAT in achieving robust segmentation results. The CNN method looks like a good way to deal with the problems of standardization and generalizability in brain tumor detection. CNNs are necessary for correctly identifying tumors because they can get all kinds of information from medical pictures. The suggested model had an accuracy of 0.9847, a mean Intersection over Union (IOU) of 0.8185, a precision of 0.9855, a sensitivity of 0.9840, a specificity of 0.9952, an edema dice coefficient of 0.2086, and a boosting dice coefficient of 0.2919. By focusing on learning invariant features across different tumor classes while preserving tumor-specific characteristics, When accurate and reliable tumor segmentation is needed for planning and tracking treatment, BraTS-GoAT shows a lot of promise for use in therapeutic settings. To help people plan their treatments and guess what will happen with their cancer, experts will soon be looking into how to mix radiomics features from segmented tumors with clinical data.

## Figures and Tables

**Fig. (1):**
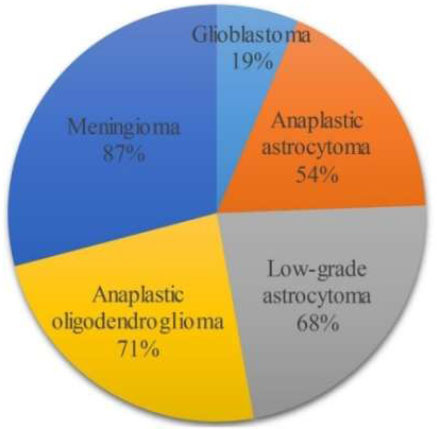
The five tumor types with their survival rates for patients aged between 20–44 [5].

**Fig. (3):**
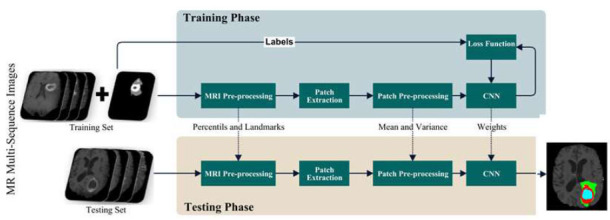
Block diagram of proposed technique.

**Fig. (4):**
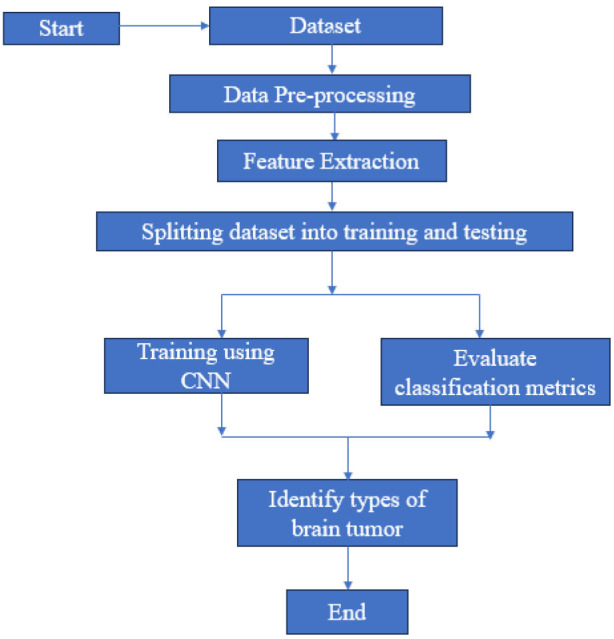
Flowchart of the proposed framework.

**Fig. (5):**
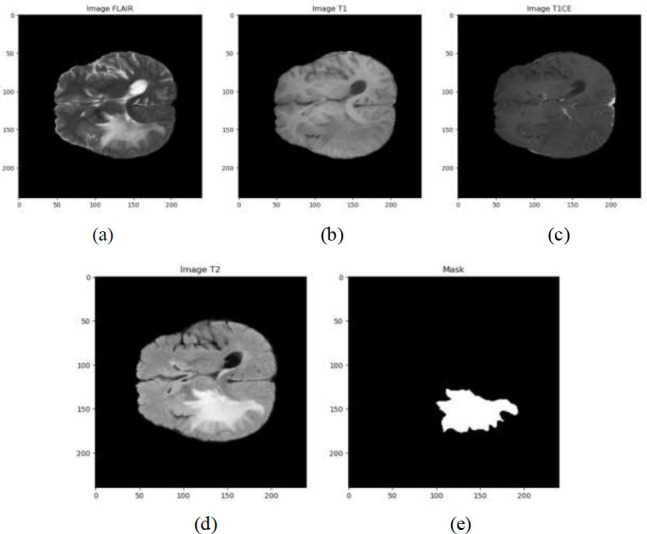
Visualization of preprocessing image (**a**) FLAIR image (**b**) Image T1 (**c**) Image T1CE (**d**) Image T2 (**e**) Mask.

**Fig. (8):**
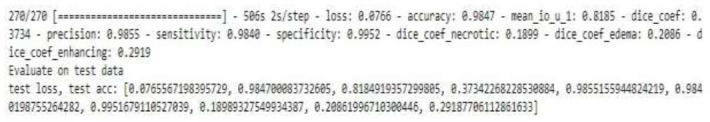
Performance metrics of testing model.

**Fig. (9):**
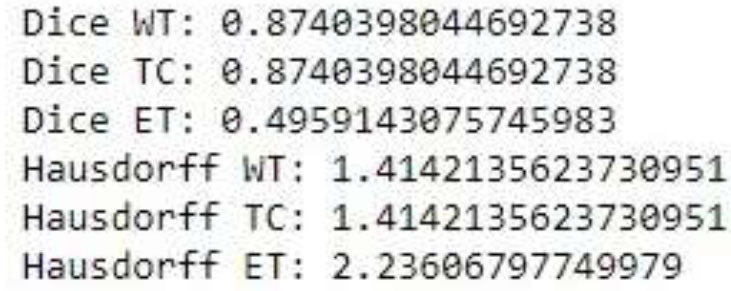
Performance metrics of the proposed method.

**Fig. (10):**
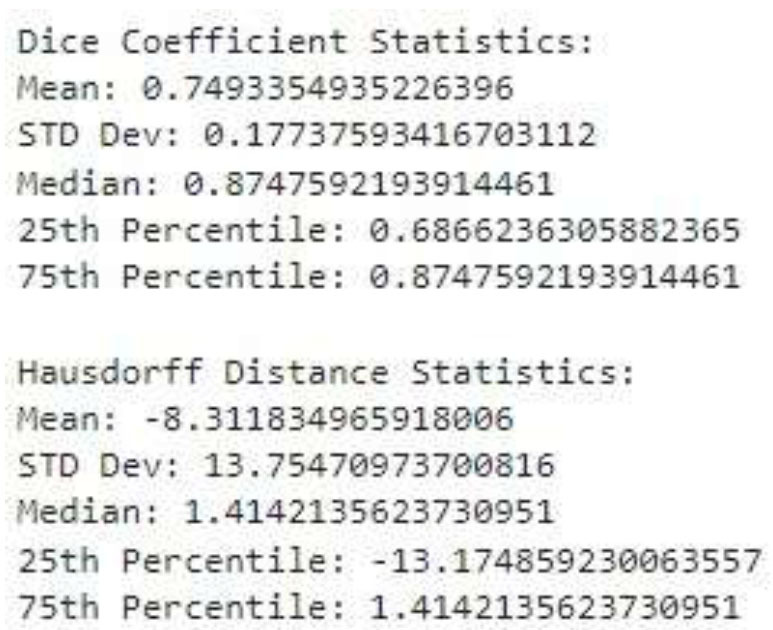
Performance of segmentation model.

**Fig. (11):**
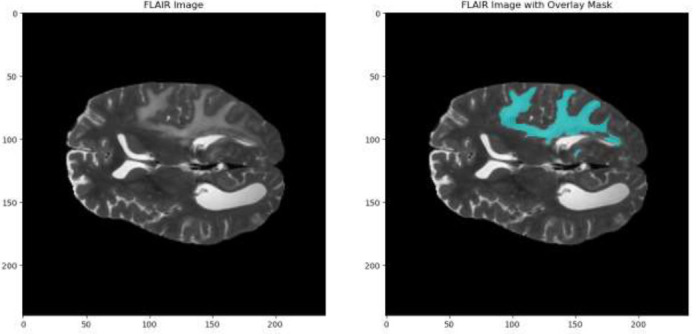
Segmentation mask of FLAIR image.

**Fig. (12):**
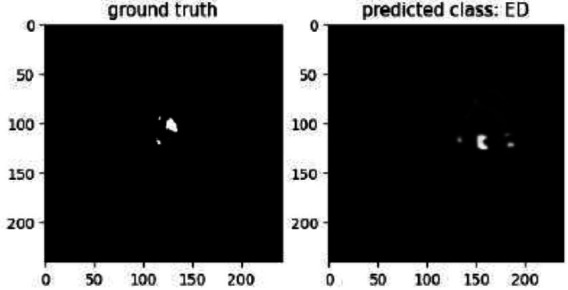
Prediction *vs* ground truth.

**Fig. (13):**
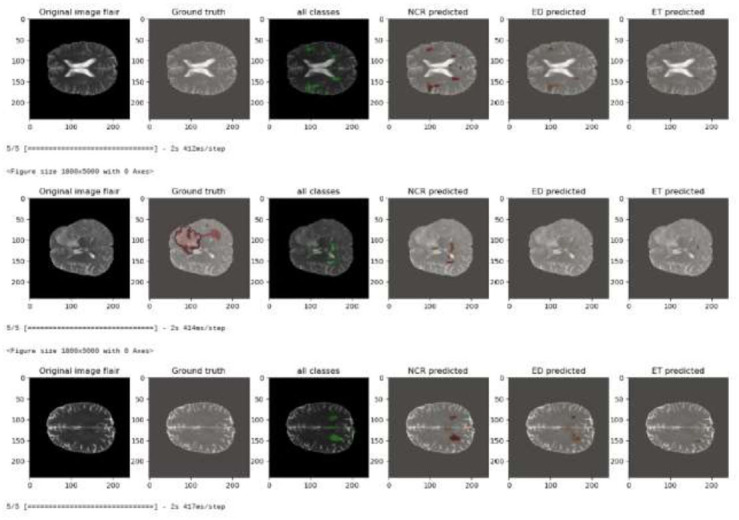
Qualitative result of the proposed framework.

**Table 1:** Comparison of reviewed technique

**Authors (Refs.)**	**Technique**	**Outcome**
**Jiajia Li., (2024)** [12]	DiffCAS	DiffCAS achieves state-of-the-art performance in terms of dice coefficients 84.41% and 84.59%, on the ASOCA dataset and ImageCAS dataset, respectively.
**Sunil *et al*., (2024) [** **13** **]**	SIENNA	The suggested method attained an accuracy of 92% in non-tumor which is higher than other methods.
**Kim *et al*., (2024) [** **14** **]**	3D-U-Net	The estimate of dropout uncertainty successfully reached the desired level of performance using the minimum amount of annotated data.
**Berkley *et al*., (2023) [** **15** **]**	3D U-Net	The recommended method got results of 0.764 for the whole tumor, 0.648 for the center of the tumor, and 0.61 for the tumor that was getting better.
**Hu *et al*., (2023) [** **16** **]**	MCN	The suggested method demonstrated superior accuracy in predicting outcomes compared to both approaches that operate across different domains.
**Koirala *et al*., (2023) [** **17** **]**	Hybrid method	The results of the study demonstrate that using an ensemble strategy, which combines many designs, produces better performance than using individual models alone, resulting in enhanced assessment metrics.
**Stember *et al*., (2022) [** **18** **]**	Deep Q learning	The Deep *Q* learning approach showed a gradual improvement in its ability to generalize to the testing set as training progressed, achieving an accuracy of 70%.
**Rawat *et al*., (2022) [** **19** **]**	RNN	When unknown data was sent to the LN-based model before normalization, the validation die scores were 0.90 for edema, 0.74 for tumors that weren't enhancing, and 0.95 for tumors that were enhancing. It's better to have these numbers than not having them normalized.
**Henry *et al*., (2021) [** **20** **]**	Deep Supervised	When tested on the last set of problems, the suggested method got a dice coefficient of 0.79, a Hausdorff distance (95%) of 20.4, a difference of 6.7 mm between the points, and a difference of 19.5 mm.
**Divya *et al*., (2021) [** **21** **]**	EMMA	One of the BraTS-challenge datasets has mean Fl-scores of 0.82 for the tumor core, 0.87 for the whole tumor, and 0.78 for enhancing tumor.
**Hua *et al*., (2020) [** **22** **]**	Cascade V-Net	The whole tumor got an average Dice score of 0.9048, the core of the tumor got an average of 0.8364, and the tumor that was getting bigger got an average of 0.7748.
**Xue *et al*., (2020) [** **23** **]**	Convolutional U-Net	Using the BraTS 2019 dataset for testing, that model got an average dice value of 0.75 for the tumor that was improving, 0.90 for the whole tumor, and 0.83 for the subregions of the tumor core.
**Wang *et al*., (2019) [** **24** **]**	CNN	Findings showed that adding test-time augmentation makes brain tumor segmentation more accurate.

**Table 2:** Classification report of model.

-	**Precision**	**Recall**	**F1-score**	**Support**
NOT Tumor	0.99	0.99	0.99	437509975
NCR	0.35	0.38	0.36	471935
ED	0.24	0.37	0.29	2907516
ET	0.67	0.30	0.42	1478574
Accuracy	-	-	0.98	442368000
Macro avg	0.56	0.51	0.52	442368000
Weighted avg	0.99	0.98	0.99	442368000

**Table 3:** The proposed model hyperparameter value.

No. of epochs	200
Batch size	5
Optimizer	Adam
Activation function	Softmax
Loss	Categorical cross-entropy
Metrics	Accuracy, MeanIoU, Dice coeff
Callback functions	Csv logger, Early stopping, Model Checkpoint

**Table 4:** Comparison of the proposed approach against some methods in the literature.

**Method (Refs.)**	**Precision**	**Accuracy**	**Dice Score wt**	**Dice Score tc**	**Dice Score et**	**HD95 wt**	**HD95 tc**	**HD95et**	**Sensitivity**	**Specificity**
Ronneberger *et al*. [27]	0.78	0.85	0.82	0.75	0.79	6.2	7.5	5.8	0.82	0.87
Çiçek *et al*. [28]	0.76	0.83	0.80	0.73	0.77	6.5	7.2	6.0	0.8	0.85
Kamnitsas *et al*. [29]	0.77	0.84	0.81	0.74	0.78	6.0	7.0	5.5	0.81	0.86
Havaei *et al*. [7]	0.75	0.82	0.79	0.72	0.76	6.8	7.5	6.2	0.79	0.84
Menze *et al*. [6]	0.79	0.86	0.83	0.76	**0.80**	5.5	6.8	5.2	0.83	0.88
Isensee *et al*. [30]	0.74	0.81	0.78	0.71	0.75	7.0	7.8	6.5	0.78	0.83
Milletari *et al*. [31]	0.77	0.84	0.81	0.74	0.78	6.2	7.0	5.7	0.81	0.86
Myronenko *et al*. [32]	0.76	0.83	0.80	0.73	0.77	6.5	7.2	6.0	0.8	0.85
Wang *et al*. [33]	0.78	0.85	0.82	0.75	0.79	5.8	6.5	5.0	0.82	0.87
Andermatt *et al*. [34]	0.75	0.82	0.79	0.72	0.76	6.8	7.5	6.2	0.79	0.84
**Proposed method**	**0.98**	**0.98**	**0.87**	**0.87**	0.49	**1.4**	**1.4**	**2.2**	**0.98**	**0.99**

## Data Availability

The material used in this experimentation is at https://github.com/vaidehisatushe/BraTS2024_MICCAI

## LIST OF ABBREVIATIONS

FC: Fully Connected
CNN: Convolutional Neural Network
BraTS: Brain Tumor Segmentation challenge
GoAT: Generalizability Across Tumors
IOU: Intersection over Union
MRI: Magnetic resonance imaging
CNs: Central nervous system
MRIs: Magnetic resonance pictures
PET: Positron emission tomography
WHO: World Health Organization
ARFE: Adaptive residual feature enhancement
ML: Machine Learning
AGI: Artificial general intelligence
MCN: Mixture of Calibrated Networks
EM: Expectation-maximization
LN: Local normalization
EMMA: Ensemble of Multiple Architectures
GLCM: Gray-Level Co-occurrence Matrix
ET: Enhancing Tumor
WT: Whole Tumor
TC: Tumor Core
FLAIR: Fluid-Attenuated Inversion Recovery
CSF: Cerebrospinal fluid
